# Pneumomediastinum and Pneumothorax Following Non-invasive Respiratory Support in Patients With Severe COVID-19 Disease

**DOI:** 10.7759/cureus.18796

**Published:** 2021-10-15

**Authors:** Juan Camilo Gutierrez-Ariza, Tómas Rodriguez Yanez, Maria Cristina Martinez-Ávila, Amilkar Almanza Hurtado, Carmelo Dueñas-Castell

**Affiliations:** 1 Critical Care Medicine, Clinica La Ermita, Cartagena, COL; 2 Critical Care Medicine, Universidad De Cartagena, Cartagena, COL; 3 Epidemiology and Public Health, Nuevo Hospital Bocagrande, Cartagena, COL; 4 Intensive Care Medicine, Universidad de Cartagena, Cartagena, COL; 5 Intensive Care Medicine, Gestión Salud IPS, Cartagena, COL

**Keywords:** sars-cov-2, covid-19, high flow nasal canula, non-invasive mechanical ventilation, pneumothorax (ptx), pneumomediastinum

## Abstract

The coronavirus disease-2019 (COVID-19) pandemic led to an increased number of patients with pneumothorax and pneumomediastinum owing to complications attributed to viral pneumonia regardless of the use of mechanical invasive ventilation and the elapsed time of infection. The pathophysiology remains unknown. However, the Macklin effect is shown as the most plausible mechanism along with possible barotrauma secondary to a high-flow nasal cannula and noninvasive mechanical ventilation. We present two cases of patients who developed pneumomediastinum and tension pneumothorax. One of the patients was studied during infection and the other after recovery. Both received appropriate and timely treatments with successful outcomes. It is important to be aware of these potentially fatal complications as early management can reduce the associated morbidity and mortality.

## Introduction

As time progresses, knowledge on the complications secondary to coronavirus disease-2019 (COVID-19) or severe acute respiratory syndrome coronavirus 2 (SARS-CoV-2) infections has been increasing. Barotrauma is damage caused to the tissues as a result of the pressure gradient between an unvented body cavity and the surrounding air/fluid interface, or across a tissue plane [1].

Pneumothorax and pneumomediastinum are predominantly seen as complications of invasive mechanical ventilation [2,3]. Although uncommonly associated with viral pneumonia, a higher incidence of barotrauma in patients with SARS-CoV-2 infection has been reported [4]. Accordingly, based on available evidence, barotrauma among patients with COVID-19 occurs in 1% of hospitalized patients and in 2% of intensive care unit (ICU) patients who have never received invasive mechanical ventilation [5,6].

Herein, we report two cases of patients infected with severe SARS-CoV-2 treated with a high-flow nasal cannula (HFNC) and noninvasive mechanical ventilation (NIMV) who developed pneumomediastinum and pneumothorax.

## Case presentation

Case 1

A 48-year-old woman with a past medical history of hypertension and morbid obesity was presented to the emergency department (ED) with symptoms of fever, diarrhea, dyspnea, and dry cough which lasted for 3 days. On admission, vital signs were observed which included tachycardia (100 beats/min), fever (38.3°C) tachypnea (24 breaths/min), and 89% oxygen saturation when she breathed freely room air. The physical examination was unremarkable. Blood tests revealed an elevated C-reactive protein, leukocytosis-lymphopenia, and arterial blood gases (ABG) showed mild hypoxemia (PaO2: 60 mmHg) with no other test abnormality. An initial chest X-ray revealed ground-glass opacifications of both lung fields. The patient received treatment (while she was awake) with sulbactam-ampicillin, hydrocortisone (used due availability), low-molecular-weight heparin, and supplementary oxygen with HFNC (as much as she could tolerate) in a prone position.

**Figure 1:**
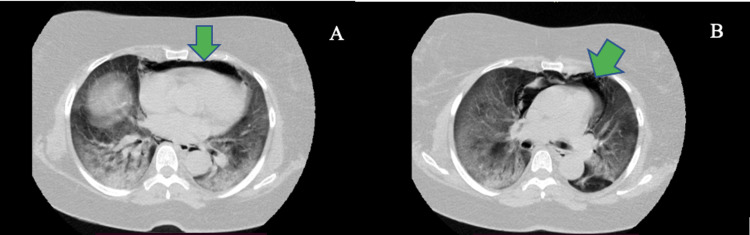
A, B Axial computed tomography image showing pneumomediastinum (green arrows).

On the third day of admission, COVID-19 infection was confirmed by reverse transcriptase-polymerase chain reaction (RT-PCR) testing, and the patient’s breathing work increased as oxygen saturation (SpO2) fell. New ABG tests were performed which showed severe hypoxemia (partial pressure of oxygen [PaO2]: 38 mmHg PaO2/fraction of inspired oxygen [FiO2] 99 mmHg). The patient received NIMV (inspiratory positive airway pressure [IPAP]=22 cmH20, expiratory positive airway pressure [EPAP]=10 cmH20) owing to a worsened respiratory distress state and was immediately transferred to the ICU. Noncontrast chest computed tomography (CT) showed multiple areas of consolidation and ground-glass opacities in lobes, the presence of pneumomediastinum, and the absence of pneumothorax (Figure 1).

Surgical consultation was sought owing to the size of the pneumomediastinum and hemodynamical stability. It was decided to continue to provide support measures based on a conservative management strategy; no variations in EPAP or IPAP were tried, and they continued at the same settings. Her respiratory symptoms improved as SpO2 levels increased up to 95% under ambient air, and the fever disappeared. She completed the treatment with no further laboratory findings. Therefore, 10 days after admission, the patient was discharged.

Case 2

This is the case of a 40-year-old male, nonsmoker, with no known comorbidities, previously diagnosed with severe COVID-19. The patient required ICU treatment with NIMV for 5 days (IPAP=12 cmH20, EPAP=7 cmH20), and 10 days of hydrocortisone treatment (used due availability). The patient recovered without complications. Seven days after discharge, the patient was readmitted to the ED owing to the deterioration of his general status, acute pleuritic pain in the left hemothorax, and sudden dyspnea symptoms. On physical examination, his blood pressure was 80/50 mmHg, heart rate was 108 beats/min, respiratory rate was 28 breaths/min, and oxygen saturation was 83%. The patient received 2 L/min of supplementary oxygen. Physical examination showed jugular vein distention and intercostal retractions.

**Figure 2:**
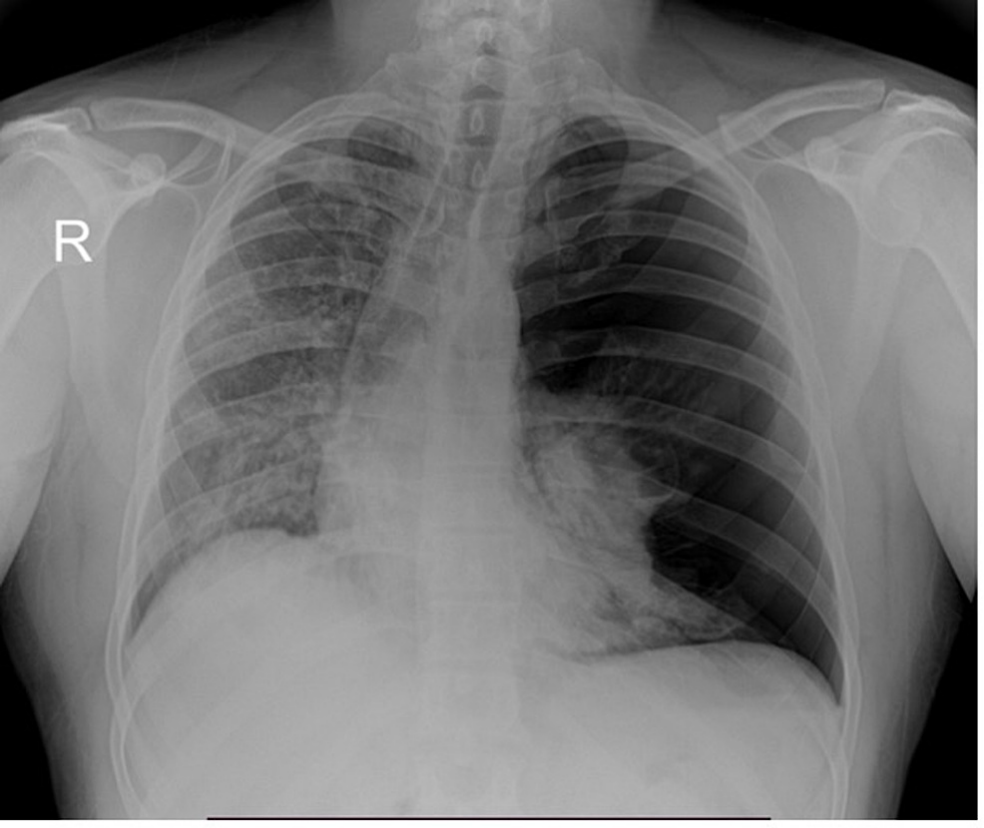
Portable chest X-ray of a large left-sided pneumothorax with mediastinal shift which required the placement of a thoracostomy tube.

After arrival, he was placed on an HFNC. An X-ray was obtained in the ED with a portable X-ray system, which demonstrated a large left-sided apical tension pneumothorax with the collapse of the left lung (Figure 2). A left thoracostomy tube was immediately placed under sterile conditions, and he was referred to the ICU respiratory service.

Fortunately, the patient responded to treatment, and he was hemodynamically stabilized. A standing chest X-ray performed on day 6 of admission after the removal of the thoracostomy tube showed a complete reexpansion of his left lung indicative of resolution of the problem. He remained stable and was discharged seven days later.

## Discussion

Pneumothorax and pneumomediastinum are defined as the states in which free air is present in the pleural and mediastinal cavities, respectively [7].

It can be precipitated by various triggers that include spontaneous onset, trauma to the thoracic cavity secondary to acute respiratory distress syndrome (ARDS), mechanical ventilation, tracheobronchial injury, cardiothoracic interventions, esophageal rupture, parturition, Valsalva maneuvers, infections, malignancy, complications during surgical procedures, and iatrogenic injuries, such as ventilator-associated barotrauma [8]. Asthma, chronic lung diseases, such as chronic obstructive pulmonary disease and interstitial lung disease, and drug abuse are some predisposing factors [8]. None of our patients were active smokers, but they both had risk factors; the first patient had excessive coughing because of the active infection and the second one had a previous acute lung injury secondary to COVID-19 pneumonia.

The exact mechanism of barotrauma in nonventilated patients remains unknown. However, the Macklin effect, described in 1944, has been proposed as a possible etiology [9] owing to the structural alterations in the lung parenchyma from COVID-19. These include surface protein disruption, such as downregulation of surfactant, loss of extracellular matrix and basement membrane, damaged type 2 pneumocytes, and hypercoagulability [10].

The Macklin effect is mainly associated with high pressure causing a rupture along the alveolar tree, which leads to an abrupt increase in the intraalveolar pressure [9]. Released alveolar air centripetally releases through the pulmonary interstitium along the bronchovascular sheaths toward the pulmonary hila, into the mediastinum leading to spontaneous pneumothorax, pneumomediastinum, subcutaneous emphysema. Sometimes it continues to diffuse through other anatomical regions [9].

The symptoms include acute retrosternal chest pain radiating to the neck or the back, subcutaneous emphysema, dyspnea, fatigue, feelings of tightness in the chest, tachycardia, hoarse voice, and even cyanosis, and emesis [7]. Our patients’ symptoms coincide with those referred to in the literature, namely, chest pain and breathlessness. We want to highlight that both diseases are potentially fatal depending on the underlying cause, extension of the injury, and comorbidities [7].

It is essential to emphasize the oxygen delivery method employed because patients receiving elevated positive end-expiratory pressure (PEEP) have a higher risk of developing lung lesions. Our patients received oxygen by HFNC and NIMV. Owing to its advantages over invasive mechanical ventilation, these approaches are considered secure for ensuring an adequate oxygen saturation level in hypoxemic patient cases. Nevertheless, they may lead to barotrauma as they could reach PEEP levels as high as 7.4 cmH20 [11].

The Macklin effect usually appears in chest X-ray or on CT and is manifested by linear air collections contiguous to the bronchovascular sheaths. Consequently, air may be present in the mediastinum. Multidetector-row CT has reported the highest diagnostic accuracy among diagnostic studies [7]. This finding has also been demonstrated in case 1 in which the pneumomediastinum was also identified on CT. In case 2, owing to the patient’s hemodynamical instability and tension pneumothorax, no CT was performed; however, it was proven necessary to conduct the emergency procedure of thoracostomy tube placement.

Finally, treatment depends on hemodynamic stability and the sizes of the pneumomediastinum or pneumothorax. Typically, symptomatic and conservative measures are adequate, including the use of oxygen-alluding to nitrogen depletion and accelerated disappearance of mediastinal gas due to a higher metabolic affinity to oxygen-painkillers, and antitussives; however, any underlying etiology should be investigated in detail [7]. The patients described in this case report received treatment according to what was reported in the literature with successful outcomes.

## Conclusions

Pneumomediastinum and pneumothorax are possible complications of COVID-19 pneumonia and are manifested as forms of barotrauma facilitated by a persistent cough and lung infection, thus causing acute decompensation that can be a negative prognostic indicator for poor outcomes. This can occur during different phases of COVID-19 even on recovered patients. Thus, a close follow-up is needed, and increased awareness of this disease is highly recommended. The natural history of disease and progression, and radiological imaging combined with physical exam findings, can help identify these lesions and determine an accurate treatment to reduce the associated morbidity and mortality.
